# Understanding well-being at work: Development and validation of the eudaimonic workplace well-being scale

**DOI:** 10.1371/journal.pone.0215957

**Published:** 2019-04-25

**Authors:** Amy L. Bartels, Suzanne J. Peterson, Christopher S. Reina

**Affiliations:** 1 Department of Management, College of Business, University of Nebraska-Lincoln, Lincoln, Nebraska, United States of America; 2 Thunderbird School of Global Management, Arizona State University, Glendale, Arizona, United States of America; 3 Department of Management, College of Business, Virginia Commonwealth University, Richmond, Virginia, United States of America; University of Lleida, SPAIN

## Abstract

Given the amount of time and effort individuals pour into work, scholars and practitioners alike have spent considerable time and resources trying to understand well-being in the workplace. Unfortunately, much of the current research and measurement focuses on workplace well-being from only one perspective (i.e. hedonic well-being rather than eudaimonic well-being) or by generalizing between workplace well-being and general well-being. In this study, we sought to integrate the workplace context into the current eudaimonic perspective to develop an 8-item measure of eudaimonic workplace well-being. Using multi-wave data, we developed and validated a reliable, two-dimensional eudaimonic workplace well-being scale (EWWS). The measure replicated over seven samples and across 1346 participants and showed strong convergent, discriminant, and predictive validity. Furthermore, we combined EWWS with an existing measure of hedonic workplace well-being and the resulting model of overall workplace well-being explained a significant amount of variance in key organizational constructs over and above existing hedonic well-being measures. Overall, the present study suggests that the EWWS is a valuable and valid measure and, when taken together with hedonic workplace well-being, captures what it means to have a holistic sense of well-being at work.

## Introduction

In today’s modern world, work life consumes a significant part of most individuals’ lives. A recent report by Gallup showed that the average workweek for full-time employees in the United States has risen to 46.7 hours, which adds up to almost a full extra day of work [1] and 34% of Americans admit to working additional hours on the weekends [2]. These additional work hours take a toll on workers, leading to burnout and work overload in 68% of full-time workers [3]. Therefore, it is not surprising that organizational researchers have invested considerable attention trying to better understand the role that work plays in an individual’s well-being [4–7].

Well-being researchers promote two complementary perspectives to understanding well-being: hedonic and eudaimonic [8]–[9], yet much of the research focusing on well-being at work has focused only on the *hedonic* perspective which revolves around happiness and an individual’s cognitive and affective evaluation of his/her life [10]. In contrast, the *eudaimonic* perspective focuses on optimal functioning and human growth and is typically referred to as psychological well-being [11]. This perspective is grounded in the notion that well-being is more than just happiness and pleasure. It occurs when individuals’ activities and mental states are authentic and congruent with their deeply held beliefs or values [9], [12]. Unfortunately, unlike hedonic workplace well-being, no measure exists to capture well-being at work from the eudaimonic perspective [13].

Instead, most researchers have painted eudaimonic well-being with a relatively broad stroke without focusing on the workplace. They have assumed that conceptualizations and measures of *general* well-being can simply be applied or re-tooled to fit the work domain. For example, a common practice when measuring eudaimonic well-being at work is to take a general well-being item (e.g., “I have aims and objectives for living”) and add the work context (e.g., “At work, I have aims and objectives for living”) Although some general well-being items may indeed be relevant at work, others may not. This represents a key limitation in the current workplace well-being literature because general well-being is limited in its ability to capture what it means to flourish at work specifically. Given the importance of social structure and embeddedness in influencing how individuals perceive their purpose and belongingness at work, we concur with other researchers that a valid measure of eudaimonic workplace well-being necessarily involves incorporating the social context [14]–[15]. To address this point, we offer a conceptualization of eudaimonic workplace well-being that incorporates the work context into current theory on well-being from the eudaimonic perspective. Specifically, we suggest that eudaimonic workplace well-being consists of two key broad components–interpersonal and intrapersonal–that together reflect what it means to have optimal functioning and growth at work.

The purpose of our study is not only to introduce a specific conceptualization of eudaimonic workplace well-being, but also to develop and validate a new measure for researchers and practitioners who want to go beyond assessing hedonic well-being. We offer three main contributions. First, we theoretically and empirically demonstrate the distinctiveness of eudaimonic *workplace* well-being from eudaimonic *general* well-being and other similar constructs. Second, we add support to the necessity of both the hedonic and eudaimonic perspective of workplace well-being by proposing and testing a comprehensive model of workplace well-being that reflects both perspectives and more fully encompasses employees’ experiences and optimal functioning within the work context. Third, we extend our understanding of the importance of eudaimonic well-being at work by exploring the predictive ability of our measure with key organizational constructs. We utilize seven samples consisting of 1346 participants to accomplish these goals.

## Theoretical development and hypotheses

### General well-being: Hedonic and eudaimonic perspectives

Well-being researchers have traditionally emphasized one of two complementary perspectives when considering individuals’ feelings of well-being in general (i.e., across life domains). The first is the hedonic perspective which is rooted in hedonism, or the idea that the good life is created by maximizing pleasure and minimizing pain [16]. Psychologists have approached the hedonic perspective of well-being by focusing on an individual’s subjective rating of happiness, judgments about the good/bad elements of his/her life [17]. The second perspective of well-being, and the main focus of this study, is the eudaimonic perspective. Eudaimonic well-being centers on individual flourishing and fulfillment of one’s potential and draws heavily on the human growth and development literature [12], [18].

The eudaimonic perspective of well-being is primarily conceptualized and measured using Ryff’s [15] construct of psychological well-being (PWB). PWB is based on an individual’s growth and fulfillment in six dimensions. The first three dimensions–self-acceptance (positive attitudes about oneself), positive relations with others (warm, trusting interpersonal relations) and autonomy (a sense of freedom from the norms governing everyday life) are drawn from self-actualization and self-determination theory [9], [15]. The latter three draw from ideas of mastery and optimal functioning–environmental mastery (ability to control and contribute to the environment), purpose in life (a sense of purpose, directedness and intentionality) and personal growth (continuing to develop one’s potential and grow as a person). Together, the six dimensions represent the eudaimonic perspective of *general* well-being.

Despite the fact that the hedonic and eudaimonic perspectives have been shown to be conceptually and empirically distinct types of well-being [17], [19], a great deal of confusion arises because both forms are typically referred to as “well-being” making them seem interchangeable. Yet, the empirical evidence shows that, although the hedonic perspective and eudaimonic perspective are highly correlated (*r* = .70), almost 50% of the population is either high on one or the other, but *not both* [20]. Thus, research that allows the different types of well-being to be interchangeable can be problematic because scholars measuring well-being using only one of the two perspectives may form an incomplete picture since what drives a person’s level of happiness (hedonic), may be very different from what enhances his/her sense of meaning/purpose (eudaimonic).

Since scholars generally agree that both the hedonic and eudaimonic perspectives are important for understanding a person’s general well-being, we argue they should be equally important to understanding an employee’s *workplace* well-being. Yet, we do not know of any previous work that differentiates eudaimonic well-being at work from eudaimonic well-being in general. Understanding work-specific eudaimonic well-being is important because, as noted by Ryff and Singer, “Well-being, construed as growth and human fulfillment, is profoundly influenced by the surrounding contexts” [21] (p. 14). Therefore, to truly capture and influence an employee’s overall well-being at work, it is important to develop a work-specific conceptualization and measure of eudaimonic workplace well-being that can complement the hedonic perspective of workplace well-being.

### Conceptualizing eudaimonic workplace well-being

We draw on the eudaimonic perspective of general well-being [15] and social context theory [14] to conceptualize eudaimonic *workplace* well-being, defined as an employee’s subjective evaluation of his or her ability to develop and optimally function within the workplace. We theorize that eudaimonic workplace well-being should encompass the six dimensions previously discussed that are associated with eudaimonic general well-being (i.e., psychological well-being) but that it also should include certain context-specific attributes. In particular, Keyes [14] advanced a social context theory of well-being that considers five dimensions of context that may be particularly influential in determining an individual’s well-being at work: social integration, social acceptance, social contribution, social actualization, and social coherence. We believe that the six dimensions of psychological well-being and the five dimensions associated with social context theory can be parsimoniously represented by two broad dimensions–interpersonal workplace well-being and intrapersonal workplace well-being. The interpersonal dimension focuses on the external or social aspects of work that shape an individual’s experience of fulfilling his/her potential and intrinsic goals while the *intra*personal dimension focuses on the internal or personal factors that encourage the same.

#### Interpersonal dimension

The interpersonal dimension of workplace well-being captures the impact of social interactions within the workplace that contribute to an individual’s ability to achieve “psychosocial flourishing” [22]–[23]. In particular, the interpersonal dimension goes beyond provisions of social support to recognize the external components of optimal functioning while also answering the call from scholars to create an understanding of well-being that takes into account how individuals’ experiences of well-being are impacted by the social structures and communities within which they exist [14]. It encompasses two components of social context theory–social acceptance and social integration–and two components of psychological well-being–self-acceptance and positive relations with others. Both social acceptance and self-acceptance are associated with positive feelings about oneself, but social acceptance focuses on the comfort that one feels from being a part of a particular context–the work environment in this case–while self-acceptance focuses solely on the maturity needed to form and reciprocate relationships [14]. Similarly, social integration and positive relations with others both focus on the relationships with others in the workplace [14]–[15]. Collectively, these four components focus on the quality relationships with those external to the individual (e.g., one’s coworkers, leaders or even customers) and we suggest they can be captured in one combined dimension representing interpersonal eudaimonic workplace well-being.

#### Intrapersonal dimension

The intrapersonal dimension of workplace well-being reflects internal feelings of value and meaningfulness within the workplace through the actual work itself or one’s personal development as a worker. In particular, it recognizes the employee’s desire for self-control at work, which has traditionally been a critical part of the eudaimonic perspective of well-being [24]. Employees more than ever before view work as more than just a paycheck. Younger workers in particular express a desire to be in a work role that allows them to have purpose and to make a difference in society [25].

The intrapersonal dimension draws on four components of social context theory–social coherence, social contribution, social actualization, and social coherence–and four components of psychological well-being–purpose in life, autonomy, environmental mastery, and personal growth. Each of these focuses inward on the energy and value that one derives from the workplace. Social coherence and purpose in life both focus on fulfilling one’s purpose in one’s environment. Similarly, social contribution and environmental mastery focus on the role an individual plays in creating, achieving, and sustaining the social context through his/her meaningful connections with others. Social actualization incorporates the social context along with autonomy and personal growth and combines it into one dimension [14]. Overall, each of these components are aligned in their focus on the personal growth and development an employee experiences and thus can be adequately captured in one combined dimension of intrapersonal eudaimonic workplace well-being. Therefore, we hypothesize that the interpersonal and intrapersonal dimensions together comprise eudaimonic workplace well-being:

*Hypothesis 1*: *The construct of eudaimonic workplace well-being consists of two distinguishable dimensions that define its domain*: *interpersonal and intrapersonal*.

### Distinguishing eudaimonic general well-being from eudaimonic workplace well-being

Accounting for the work-specific context in well-being research is essential given that certain aspects of well-being may manifest themselves differently inside versus outside the workplace. Scholars focusing on the hedonic perspective have been quick to recognize this distinction. These researchers clearly differentiate general well-being from workplace well-being (i.e., job satisfaction) and emphasize the need for both constructs [17], [26]. Empirical evidence also supports the need for both constructs by demonstrating only a moderate correlation between hedonic general well-being and job satisfaction (*r* = .44) [19]. Furthermore, researchers have pointed out that the outcomes and antecedents of hedonic general well-being and job satisfaction are very different. Following a bandwidth fidelity argument [27], general well-being is more likely to be associated with more global individual differences that match the breadth of the construct such as socioeconomic status [28], while job satisfaction is more apt to be related to work behaviors such as job performance [29] and the leadership style of one’s supervisor [30]. Therefore, similar to the hedonic perspective, we hypothesize that general eudaimonic well-being will be correlated, yet empirically distinct from eudaimonic workplace well-being.

*Hypothesis 2*: *Eudaimonic workplace well-being is distinct from general eudaimonic well-being*.

### Discriminating eudaimonic workplace well-being from other constructs

Given the lack of a comprehensive conceptualization of eudaimonic well-being within the workplace, scholars have been forced to use different proxy constructs. Employee engagement has been used as both a measure of the eudaimonic general well-being and even eudaimonic workplace well-being [11], [31]. Employee engagement is a motivational process where an individual harnesses his/her physical, cognitive, and emotional energy into his/her role performance [32]. Although both eudaimonic workplace well-being and employee engagement focus on energy and positive psychological states, engagement focuses more on the process of putting forth such energy whereas eudaimonic workplace well-being focuses on the psychological satisfaction that occurs when such energy leads to positive functioning.

Life satisfaction is another construct often used to reflect the hedonic perspective of general well-being [33]–[34]. It focuses on a broad conceptualization that allows individuals to assess their happiness with their lives as a whole [35], whereas eudaimonic workplace well-being emphasizes growth and development. Scholars suggest that an individual may be happy when experiencing growth and development, but not *always* [9]. Therefore, similar to employee engagement, we expect life satisfaction and eudaimonic workplace well-being to be correlated, yet distinct.

We also theorize about the convergent and discriminant validity of two additional constructs because of their conceptual overlap with the interpersonal dimension of eudaimonic workplace well-being. In particular, social undermining and leader-member exchange should be significantly related, yet distinct from eudaimonic workplace well-being. Social undermining occurs when an employee experiences a threat to his/her sense of belonging due to the negative actions of his/her coworkers [36]. Leader-member exchange is the quality of relationship that an employee has with his/her leader. Within the workplace, there is a need to relate to and connect with others in the workplace through coworker and leader relationships [37], so these feelings of belongingness should be highly correlated to eudaimonic workplace well-being. However, the interpersonal dimension of eudaimonic workplace well-being not only examines the feelings of belongingness and connectedness, but also captures specifically how these interactions create personal flourishing. Thus, eudaimonic workplace well-being likely is an outcome of both social undermining and leader-member exchange relationships and, therefore, should represent a distinct construct.

*Hypothesis 3*: *Eudaimonic workplace well-being is significantly related*, *yet distinct from other key constructs such as (a) employee engagement*, *(b) life satisfaction*, *(c) leader-member exchange*, *and (d) social undermining*.

### Predictive validity of eudaimonic workplace well-being

Conceptualizing and measuring eudaimonic workplace well-being is critical because an employee’s level of eudaimonic workplace well-being is predictive of key attitudes and behaviors within the workplace. Specifically, theory suggests that positive functioning should be closely related to creativity and innovation [38]–[39]. Drawing on self-determination theory, scholars have suggested that achieving higher levels of well-being as children leads them to be more intrinsically motivated and more creatively engaged later in life than individuals who had lower levels of well-being as children [39]. Thus, we hypothesize that eudaimonic workplace well-being will be positively related to an individual’s level of creativity within the workplace.

We also predict that eudaimonic workplace well-being will be significantly related to an employee’s withdrawal behaviors such as turnover intentions and absenteeism. An individual’s level of eudaimonic workplace well-being is reflective of the individual’s level of functioning and the resources they have available. Based on the conservation of resources theory (COR) [40], we suggest that an employee with depleted resources is more likely to demonstrate more frequent absenteeism and have a higher desire to leave his/her current job. Furthermore, prior work demonstrates relationships between withdrawal and similar well-being constructs such as job satisfaction [41] and employee engagement [42]. Therefore, we hypothesize that eudaimonic workplace well-being will be negatively related to both turnover intentions and absenteeism.

*Hypothesis 4*: *Eudaimonic workplace well-being is positively related to (a) employee creativity and negatively related to (b) employee turnover intentions and absenteeism*.

### Integrating eudaimonic and hedonic workplace well-being

As noted earlier, the two perspectives of well-being are best seen as complementary concepts rather than substitutable ones, so a comprehensive conceptualization of overall workplace well-being should incorporate both perspectives. Empirical evidence supports this notion. For example, Nix, Ryan, Manly, and Deci [43] also conducted a series of experiments and found that an individual who had success at the same activity in which s/he had little choice in the tactics taken concurrently reported high levels of hedonic well-being and low levels of eudaimonic well-being. In addition, recent neurological evidence suggests different antecedents for each perspective of well-being. Whereas goal-directed behavior was significantly related to the eudaimonic perspective of general well-being (*r* = .21*)*, it was not related to the hedonic perspective [44]. Therefore, we suggest that understanding and measuring *both* hedonic and eudaimonic workplace well-being is just as critical as it is for understanding and measuring general well-being. In particular, we propose that the intrapersonal and interpersonal dimensions can be combined with hedonic workplace well-being to comprehensively and parsimoniously capture overall workplace well-being.

*Hypothesis 5*: *The best model fit for workplace well-being is a three-dimensional model consisting of the dimensions of eudaimonic perspectives of workplace well-being and hedonic perspective of workplace well-being*.

Finally, we acknowledge that, in order for a model of overall workplace well-being to be both useful and valuable, it must be able to provide information about key organizational constructs that the hedonic perspective alone cannot provide [45]. In particular, we suggest that overall workplace well-being can predict individual organizational citizenship behaviors (OCBs) over and above the hedonic perspective, and even help determine whether, as suggested previously by some scholars, the relationship between an employee’s workplace well-being and OCBs is driven by trait affect [46]. Organizational citizenship behaviors are acts individuals do that aid others that are not part of the employee’s “official role” [47]–[48]. Scholars have suggested an overall relationship between the hedonic perspective of workplace well-being and organizational citizenship behaviors, but the relationship may be driven by a third variable related to both such as trait affect. We suggest that the driving force in these relationships is eudaimonic workplace well-being (once we control for trait affect) rather than the hedonic perspective of workplace well-being.

We also suggest that our broader conceptualization of workplace well-being can explain additional variance in the relationship between workplace well-being and employee popularity beyond individual differences in trait affect. Defined as being generally accepted by one’s coworkers in the organization [49], popularity has been previously linked to overall job satisfaction [50]. We suggest that overall workplace well-being will explain significant variance in an employee’s feelings of popularity since the interpersonal dimension of eudaimonic workplace well-being reflects a sense of belonging and integration. Although popularity is not always seen as a key organizational construct, recent work suggests popularity may be key to a functioning workplace as employee popularity can lead to perceptions of justice [51] and decreased counterproductive work behaviors [49].

*Hypothesis 6*: *Overall workplace well-being is significantly related to key organizational constructs such as organizational citizenship behaviors and employee popularity even when controlling for trait affect*.

## Methods

We sought to create and validate the Eudaimonic Workplace Well-being Scale (EWWS) to allow scholars to isolate and study an employee’s workplace well-being specifically from the eudaimonic perspective. To begin, we created the EWWS by generating and refining the final items included in the scale. Next, we validated the new scale by assessing the psychometric properties of the scale including reliabilities and factor structure, and demonstrated convergent and discriminant validity. We also examined the scale’s predictive validity to begin mapping the nomological network of eudaimonic workplace well-being. Finally, we tested whether eudaimonic workplace well-being could be combined with the hedonic perspective to reflect overall workplace well-being and tested the scale’s predictive ability. All data was collected in compliance with the Institutional Review Board at Arizona State University (IRB: STUDY00001649). None of the samples listed below included any minors and/or special populations and the data was analyzed anonymously. Therefore, the need for consent was waived by the Institutional Review Board during the review process.

### Item generation and refinement of EWWS

First, we generated potential items using two key approaches. We used a deductive approach to consider similar theoretical constructs to workplace well-being in order to derive potential items [45]. We examined and selected items from constructs capturing overall well-being and those that appeared similar to eudaimonic workplace well-being such as: subjective well-being, psychological well-being, burnout, irritation, self-rated anxiety, vitality, promotion-focus, engagement, belongingness, trust, commonality with others, perception of kindness in others, and social integration. Then, we revised the items to make sure they specifically referenced the workplace.

Second, we conferred with an expert in the well-being field to help generate additional items for each dimension to create one scale reflecting the two dimensions of eudaimonic workplace well-being. This process resulted in an initial set of 36 items with 25 intrapersonal items and 11 interpersonal items. Circling back to our theory, the intrapersonal items focused on energy, purpose, personal growth, and the ability to contribute to the workplace; similarly, the interpersonal items focused on comfort, the ability to form and reciprocate relationships with others in the workplace. To create a comprehensive yet parsimonious scale, we sought to reduce the number of items while maintaining the bandwidth of the construct. We used an item-sort task to assess the content validity of the scale and exploratory factor analysis (EFA) to further reduce the number of items [52].

For the item-sort task, we recruited 44 participants through friends and family member recommendations from academic colleagues, following the steps outlined by Hinkin and Tracey [53]. To assess content validity of each dimension of the scale, we provided respondents with all scale items and the definition of one of the dimensions. Respondents indicated how well each item fit with the definition on a 1–5 Likert scale, then we used the results to determine which items most individuals strongly agreed reflected each of the three dimensions. Next, we utilized 120 MBA students from a Southwestern university for EFA using principal axis factoring with a varimax rotation. Examining the scree plot, we extracted two factors. The intrapersonal factor accounted for the most variance at (49.3%) whereas the interpersonal factor accounted for at (14.8%) of the variance. We eliminated all items with factor loadings of less than .70 on their primary factor and more than .30 on the other factor to create four items for each dimension. The results were an 8-item scale with four items reflecting each dimension (see Appendix for full list of items). Each dimension showed strong internal reliability (*r* = .91 and *r* = .86 for interpersonal and intrapersonal respectively) and the overall scale showed strong reliability as well (*r* = .89).

### Construct validation of EWWS and predictive validity of overall well-being

To examine the construct validity of the EWWS and our specific hypotheses, we utilized multiple samples and multiple time periods [45]. Following the guidelines set by Ferris, Brown, Berry, and Lian [54], we used five separate samples (Samples 3/4 through 7) to test the EWWS across a variety of environments and situations [55] (see Table 1). Specifically, we used all five samples to test model fit, psychometric properties and convergent and discriminant validity. We used Sample 6 to assess the predictive validity of EWWS and Sample 7 to assess the validity of the overall workplace well-being model. Unless otherwise noted, all items within the surveys were measured on a Likert scale (1 = strongly disagree, 5 = strongly agree).

**Table 1:** Sample descriptions.

	Participants	Industry	% of female respondents	Median age range	Average tenure (organization)
Sample 3^a^	98 (65% response rate)	Medical	67%	35–39 years	5.0 years
Sample 4^a^	237	Amazon Mturk	38%	35–39 years	No response
Sample 5^b^	203 (51% response rate)	Government agency	85%	30–34 years	6.23 years
Sample 6	155 (63% response rate)	Non-profit/ Insurance	74%	40–49 years	8.63 years
Sample 7	533	Amazon Mturk	34%	30–34 years	No response

^a^Sample 3 and 4 were combined because Sample 3 was not large enough to test our models. We controlled for sample number when conducting the analyses.

^b^Participants completed survey 1 online and then completed survey 2 approximately two weeks later.

#### Eudaimonic workplace well-being

We measured eudaimonic workplace well-being using the 8-item measure generated in the previous section. As noted earlier, the interpersonal dimension focuses on the comfort an individual feels at work (captured in item #1), the presence of relationships with others (captured in items #2 and #3) and the ability to form and reciprocate relationships at work (captured in item #4). The intrapersonal dimension focuses on an individual’s energy (captured in item #5), purpose (captured in item #6), creation of value (captured in item #7), and personal growth (captured in item #8).

#### Psychological well-being

We used Ryff’s [56] 18-item measure to assess the eudaimonic perspective of general well-being (α = .82). Participants were asked to rate their level of agreement with questions about their life in general (e.g., “I have goals in life and a sense of directness”).

#### Employee engagement

We used the Rich, LePine and Crawford’s [57] measure of employee engagement. Sample item includes “I exert full effort in my job” (α = .92).

#### Life satisfaction

We used Diener, Emmons, Larsen, and Griffin’s [58] 5-item measure to reflect the hedonic perspective of general well-being (α = .90). Participants were asked to rate their level of agreement with questions about their life in general (e.g., “I am satisfied with my life”).

#### Social undermining

We used the Duffy and colleagues [36] 12-item measure of coworker social undermining. Participants were asked how frequently they experience social undermining behaviors from their workers (e.g., “Spread rumors about you”; α = .94) on a 5-point scale (1 = never, 5 = always).

#### Leader-member exchange

We measured leader-member exchange (LMX) using the Liden and Maslyn’s [59] 12-item measure of LMX because it is designed to capture the multidimensionality of leader-member relationships. A sample item included “I am willing to apply extra effort, beyond that normally required, to meet my supervisor’s work goals” (α = .93).

#### Creativity

We measured employee creativity using the Oldham and Cummings’ [60] 3-item measure. After defining key terms for participants, we asked them to rate their level of creativity at work (e.g., “How original and practical is your work?”; α = .88).

#### Turnover intentions

We measured turnover intentions with the Hom and Griffeth’s [61] 3-item measure. Participants were asked about their withdrawal cognition by indicating their propensity to remain in the organization (e.g., “I expect to leave my job for another within the next year”; α = .92).

#### Absenteeism

We measured absenteeism using a 2-item measure following the recommendations of Scott and Taylor [62]. First, we asked the individual how frequently s/he is absent from work and then the average duration of such absences (α = .77). Participants rated items on a 1–5 scale ranging from (1 = zero, 5 = five or more absences).

#### Job satisfaction

Organizational scholars theorize that hedonic well-being in the workplace is best represented by job satisfaction because it represents a multi-faceted construct that incorporates both affective and evaluative facets consistent with how general hedonic well-being has been conceptualized and measured [26], [63–66]. As such, we use job satisfaction to reflect the hedonic perspective at work and integrate it with the EWWS to reflect overall workplace well-being. We measured overall job satisfaction using the Hackman and Oldman [67] measure of job satisfaction (α = .87). Participants were asked to assess their level of satisfaction with their job by indicating their level of agreement with work-specific statements (e.g., “Generally speaking, I am very satisfied with my job”).

#### Organizational citizenship behaviors

We used the Williams and Anderson’s [68] measure of organizational citizenship behaviors to best reflect the two dimensions distinguished by the target of the behaviors; either the other individuals in the organization (OCBI, α = .90) or the organization as a whole (OCBO, α = .72). In addition, we also used the overall measure of organizational citizenship behaviors (α = .86). A sample item for OCBI included “I help others who have heavy workloads” and a sample item for OCBO included “I give advance notice when I am unable to come to work.”

#### Popularity

We used the Scott and Judge’s [49] measure of popularity, but we revised the scale to be a self-report measure. Sample items included “I am well-known by my coworkers” and “I am viewed fondly by my coworkers” (α = .90).

#### Control variables

Previous work has suggested that affect may be a component of general well-being [69]. Because affect often considered part of the hedonic perspective [63], we controlled for positive and negative affect when assessing the predictive validity of the EWWS. We measured trait affect using Watson, Clark and Tellegen’s [70] 20-item measure (e.g., “interested” for positive affect; α = .90 and “distressed” for negative affect; α = .91). In addition, because all of our measures are self-report, we mitigated common method bias by measuring and controlling for social desirability bias [71]. We used Paulhus’s [72] measure of social desirability (e.g., “I am a completely rational person;” α = .68).

#### Factor structure of EWWS

To assess the factor structure of the new EWWS, we performed a confirmatory factor analysis (CFA) for each sample. Since we theorized a priori that eudaimonic workplace well-being is a higher order latent construct comprised of two underlying factors, we compared it to a baseline model in which all of the items were loaded on a single dimension. To allow the model to be identified and therefore test the two-factor model, we set the coefficients of the first-order loadings to be equal to each other [73]. The model was determined to be a good fit if the CFI is .95 or higher and the SRMR was .08 or lower [74].

The data provided strong support for the two-dimensional factor structure hypothesized a priori. For each sample, the CFI values ranged from .96 to .99 and the SRMR values ranged from .025 to .077 for the two-dimensional structure. In contrast, CFI values ranged from .68 to .82 and the SRMR values ranged from .11 to .16 for the unidimensional structure. In addition, the chi-square for the two-dimensional structure (*X*^2^ = 39.25 to 84.24, *df* = 18) indicated a better fit than the unidimensional model (*X*^2^ = 453.41 to 838.82, *df* = 20). Finally, the factor loadings on each dimension were strong ranging from .54 to .86 for the intrapersonal dimension, and from .70 to .96 for the interpersonal dimension with an average factor loading of .82 across all three samples. These loadings all exceeded the conventional cutoff of .40 [74].

We also examined the reliabilities of each dimension and the reliabilities of the scale. The coefficient alpha reliability estimates for the overall scale ranged from .87 to .90, from .83 to .85 for the intrapersonal dimension, and .87 to .93 for the interpersonal dimension. Across the samples, the average corrected item-total correlation ranged from .25 to .69 with an average item-total correlation of .50. In addition to the strong item-total correlation, all inter-item correlations were positively inter-correlated across all four samples. Altogether, the improvement of model fit for a two-factor model when compared to a one-factor model and the strong interrelationships among the two dimensions support the two-dimensional structure [75]; therefore, Hypothesis 1 is supported.

#### Convergent and discriminant validity of EWWS

We examined whether the EWWS was highly correlated, yet distinct from similar constructs to assess convergent and discriminant validity [76]. Table 2 contains the descriptive statistics and correlations for all of the constructs of which we examined potential convergent and discriminant validity. First, we examined the zero-order correlations between the proposed related constructs and eudaimonic workplace well-being as well as the pattern of intercorrelations within eudaimonic workplace well-being and the correlations between each of the dimensions and the related constructs. Convergent validity is demonstrated by strong, significant correlations between the eudaimonic workplace well-being and the proposed related constructs [76]–[77]. We found that psychological well-being (*r* = .44, *p* < .01), employee engagement (*r* = .49, *p* < .01), life satisfaction (*r* = .49, *p* < .01), social undermining (*r* = -.32, *p* < .01), and leader-member exchange (*r* = .49, *p* < .01) were all significantly correlated with the EWWS. Discriminant validity is demonstrated if the correlation between the dimensions of our scale is stronger than the correlation between either of the dimensions and any of the constructs previously found to have convergent validity with our new scale [77]. As shown in Table 2, the correlations between those six constructs and EWWS was *not* greater than the correlation between the intrapersonal and the interpersonal dimension (*r* = .50, *p* < .01), demonstrating sufficient discriminant validity.

**Table 2:** Descriptive statistics and correlations for convergent/discriminant validity of EWWS.

	Mean	Std Dev	1	2	3	4	5	6	7	8	9	10
**1. EWWS (T1)**	3.73	0.42										
**2. EWWS (T2)**	3.81	0.29	0.87									
**3. Intrapersonal (T1)**	3.77	0.63	0.87	0.80								
**4. Intrapersonal (T2)**	4.01	0.41	0.80	0.91	0.87							
**5. Interpersonal (T1)**	3.70	0.67	0.80	0.62	0.48	0.43						
**6. Interpersonal (T2)**	3.74	0.49	0.67	0.77	0.46	0.51	0.75					
**7. Psychological well-being**	3.49	0.12	0.44	0.38	0.43	0.37	0.28	0.23				
**8. Life satisfaction**	3.28	0.84	0.53	0.45	0.47	0.42	0.36	0.28	0.42			
**9. Social undermining**	1.77	0.40	-0.37	-0.28	-0.29	-0.28	-0.32	-0.19	-0.14	-0.18		
**10. Leader-member exchange**	4.04	0.43	0.50	0.47	0.44	0.45	0.39	0.37	0.20	0.24	-0.40	
**11. Engagement**	4.09	0.27	0.49	0.41	0.32	0.33	0.38	0.29	0.19	0.26	-0.18	0.23

Note: We measured eudaimonic workplace well-being over two time periods to assess stability of the measure. When comparing the intercorrelations, we used an average of the correlation for each construct.

Note: All values below -0.15 and above 0.15 are statistically significant (*p* < .05)

Second, we examined a series of three-factor models in which we set each of the two dimensions of eudaimonic workplace well-being and the related construct under a higher-order latent factor. We then tested whether this model had a better fit than a two-factor model (in which the two dimensions loaded on eudaimonic workplace well-being as described in the previous section and the correlated construct was allowed to covary freely). Next, we examined the chi-square value and Bayesian Information Criterion (BIC) [78] for each model to determine which model had a better fit for the related construct in question [79]–[80]. The results provided in Table 3 indicate that the two-factor model (eudaimonic workplace well-being as a higher order latent factor with two dimensions and the related construct as the separate construct) provided a significantly better fit than the three-factor model for all of the related constructs.

**Table 3:** Convergent/Discriminant validity of EWWS.

	Two-Factor Model			Three-Factor Model				
Variable Name	X^2^	*df*	BIC	X^2^	*df*	BIC	Avg. Squ. Factor Loading	Shared Variance with EWWS
**Psychological well-being**^**a**^	833.85	291	26300.15	877.73	291	26344.03	0.87	0.53
**Engagement**^**a**^	1019.25	429	18068.19	1042.42	429	18091.36	0.80	0.25
**Life satisfaction**	156.23	63	15892.70	155.86	62	15898.85	0.80	0.41
**Social undermining**	363.39	187	15149.62	362.71	186	15155.46	0.75	0.23
**Leader-member exchange**	611.82	168	14946.22	611.66	167	14952.58	0.75	0.44

^a^Each of these constructs is theorized as a higher order latent factor with dimensions. Therefore, rather than just allowing the construct to covary as we did with the other four constructs, we modeled it as a higher-order latent construct. For the two-factor model, we allowed the two higher-order latent constructs to covary. For the three-factor model, we actually placed all of the dimensions under a one latent factor. This allows us to compare whether the two dimensions constructs (e.g., eudaimonic workplace well-being and psychological well-being) are best seen as reflecting one higher-order latent construct or whether they are distinct.

Note: We measured eudaimonic workplace well-being over two time periods to assess stability of the measure. When comparing the different models, the results did not change whether the measure from Time 1 or Time 2 was used. Therefore, for the sake of parsimony, the results for Time 1 are reported in the convergent/discriminant analyses.

We followed Fornell and Larcker’s [80] suggestion to examine the models to determine if the average factor loadings indicate that eudaimonic workplace well-being is a separate construct. Table 3 also shows that each of the five constructs are distinguishable from the EWWS. Together, these results suggest that eudaimonic workplace well-being is indeed distinguishable from related constructs, yet demonstrates the pattern of expected relationships with related constructs.

#### Predictive validity of EWWS

Table 4 contains the descriptive statistics and correlations for all of the constructs used to test the predictive validity of our new scale. We tested each hypothesis using hierarchical linear regression using data collected at two points in time (two weeks apart) in order to better capture the predictive ability of EWWS on key constructs (Table 5). Hypothesis 4 examined potential impacts of the EWWS on key organizational outcomes such as employee creativity and employee withdrawal. Even when controlling for social desirability, negative affect and positive affect, the EWWS is significantly related to employee creativity (*β* = .26, *p* < .05), thus supporting Hypothesis 4a. Hypothesis 4b was partially supported. We found that the EWWS is negatively and significantly related to an employee’s turnover intentions (*β* = -.39, *p* < .05), however it is not significantly related to employee’s absenteeism (*β* = .091, *p* = .49). We asked individuals to give specific information about absenteeism for the two weeks between the first data collection period and the second. Given the other factors that play into an employee’s level of absenteeism (i.e., previously planned vacations or work events), a longer period may be needed to accurately capture absenteeism.

**Table 4:** Descriptive statistics and correlations for predictive validity of EWWS.

Variable	Mean	Std. Dev.	1	2	3	4	5	6	7	8
**1. Social desirability**	3.26	0.81								
**2. Positive affect**	3.51	0.66	0.45*							
**3. Negative affect**	1.74	0.57	-0.14*	-0.23*						
**4. Interpersonal dimension**	3.70	0.82	0.23*	0.39*	-0.22*					
**5. Intrapersonal dimension**	3.77	0.79	0.41*	0.65*	-0.33*	0.48*				
**6. EWWS**	3.73	0.69	0.38*	0.61*	-0.32*	0.86*	0.86*			
**7. Creativity**	3.70	0.70	0.36*	0.42*	-0.03	0.28*	0.45*	0.42*		
**8. Turnover intentions**	1.92	0.92	-0.33*	-0.43*	0.35*	-0.40*	-0.51*	-0.52*	-0.26*	
**9. Absenteeism**^**a**^	3.50	0.35	-0.16	-0.40*	0.20	-0.07	-0.25*	-0.18	-0.04	0.17

^a^Categorical variable

*p < .05

**Table 5:** Predictive validity of EWWS.

	Creativity		Turnover intentions		Absenteeism	
	Model 1	Model 2	Model 1	Model 2	Model 1	Model 2
	β	β	β	β	β	β
**Social desirability**	0.22*	0.21*	-0.14	-0.13	0.04	0.04
**Negative affect**	0.10	0.13	0.25*	0.20*	0.11	0.12
**Positive affect**	0.34*	0.18	-0.30*	-0.06	-0.39*	-0.44*
**EWWS**		0.26*		-0.39*		0.09
***R***^**2**^	0.22	0.25	0.26	0.35	0.17	0.17
***F***	8.47	4.64	10.78	11.75	5.42	0.49
**Δ*R***^**2**^		0.04*		0.09*		0.01

*p < .05

#### Factor structure of overall workplace well-being

Next, we wanted to assess the fit of the EWWS within the broader construct of overall workplace well-being. To do this, we compared a three-factor model with a one-factor model where all of the items loaded on one higher order factor of overall workplace well-being. We also compared the three-factor model with the two-factor model consisting of two separate yet correlated workplace well-being constructs–job satisfaction and the EWWS. This is a similar method as was used to determine whether the multiple dimensions of self-concept [81], general mental ability [82], and justice [83] should be modeled as individual constructs or as higher-order latent constructs.

As shown in Table 6, our results indicate that a three-factor model fit the data better than a one-factor model. The fit for the two-factor model was very similar to the three-factor model. Because chi-square comparisons between non-nested models is not appropriate, we used the BIC to directly compare models [78]. We examined the BIC for each model across all four samples and determined that, in every case, the three-factor model demonstrated a lower BIC than the two-factor model. This supports our theorizing that both the hedonic perspective and the eudaimonic perspective are distinct components of workplace well-being *and* that the intrapersonal dimension and the interpersonal dimension are distinct components as well. Finally, for the three-factor model, the factor loadings for each dimension ranged from .71 to .93 for job satisfaction, .70 to .95 for the interpersonal dimension and .54 to .86 for the intrapersonal dimension; all of which exceed the suggested cutoff of .40 [45]. Together, this evidence supports Hypothesis 5 and the notion that overall well-being is best modeled as a three-factor construct reflecting overall job satisfaction from the hedonic perspective and both the interpersonal and intrapersonal dimensions from the eudaimonic perspective.

**Table 6:** Model fit comparison of overall workplace well-being.

	**Three-Factor Model**			
	**X**^**2**^ **(*df*)**	**CFI**	**SRMR**	**BIC**
Samples 3 and 4*	99.14 (51)	0.98	0.03	8555.88
**Sample 5**	116.05 (41)	0.96	0.07	7057.96
**Sample 6**	134.84 (41)	0.98	0.04	15390.20
**Sample 7**	90.21 (41)	0.99	0.03	13880.09
	**Two-Factor Model**			
	**X**^**2**^ **(*df*)**	**CFI**	**SRMR**	**BIC**
Samples 3 and 4*	110.36 (52)	0.98	0.06	8564.29
**Sample 5**	130.59 (42)	0.95	0.09	7066.67
**Sample 6**	134.86 (42)	0.98	0.04	15393.70
**Sample 7**	97.68 (42)	0.99	0.05	13881.30
	**One-Factor Model**			
	**X**^**2**^ **(*df*)**	**CFI**	**SRMR**	**BIC**
Samples 3 and 4*	965.79 (54)	0.64	0.13	9405.10
**Sample 5**	538.00 (44)	0.74	0.10	7462.41
**Sample 6**	1415.64 (44)	0.67	0.11	16651.45
**Sample 7**	1267.02 (44)	0.71	0.11	15038.07

*Samples 3 and 4 were combined to combat sample size issues; the sample was dummy-coded and used as a control variable during the analyses

#### Predictive validity of overall workplace well-being

We also examined the predictive validity of overall workplace well-being to key organizational constructs. Theoretically, we suggest that the incorporation of the EWWS is necessary because overall workplace well-being explains more variance than job satisfaction alone and we sought to empirically test this assertion. We conducted our analyses using two separate path analyses in Mplus v.7 [84]—one examining the predictive validity of job satisfaction and one examining the predictive validity of overall workplace well-being to determine whether each construct related to each of the key organizational outcomes.

Some scholars have suggested that organizational citizenship behaviors should be separated out by the target of the behaviors–either other individuals (OCBI) within the organization or the organization (OCBO) as a whole [68], and others have suggested it is best model to model it as a higher order latent construct that reflects all targets of the behaviors (OCB) [85]. Therefore, to best compare the relationship of job satisfaction and overall workplace well-being to organizational citizenship behaviors, we tested it both ways—separately (i.e., OCBO and OCBI) and as a latent construct (OCB).

The descriptive statistics and correlations examining predictive validity of overall workplace well-being compared to job satisfaction are shown in Table 7. Overall workplace well-being was significantly related to overall OCB (*β* = .29, *p* < .01), OCBI (*β* = .33, *p* < .01), OCBO (*β* = .11, *p* < .05) and popularity (*β* = .25, *p* < .01). In contrast, job satisfaction was only significantly related to overall OCB (*β* = .14, *p* < .01) and OCBI (*β* = .18, *p* < .01) and was not significantly related to OCBO (*β* = .04, *p* = .46) or popularity (*β* = .08, *p* = .08). Together, these results provide partial support for Hypothesis 6 and suggest that it is vital to account for *both* the hedonic and eudaimonic perspectives of workplace well-being when measuring the predictive validity of well-being within the workplace.

**Table 7:** Descriptive statistics and correlations for predictive validity of overall workplace well-being.

Variable	Mean	Std. Dev.	1	2	3	4	5	6	7	8	9
**1. Positive affect**	3.30	0.71									
**2. Negative affect**	1.89	0.64	-0.24								
**3. Workplace well-being**	3.48	0.86	0.60	-.36							
**4. Intrapersonal dimension**	3.57	0.91	0.59	-0.28	0.87						
**5. Interpersonal dimension**	3.47	1.00	0.44	-0.27	0.73	0.55					
**6. Job satisfaction**	3.44	1.02	0.53	-0.35	0.94	0.75	0.50				
**7. OCB (combined)**	3.82	0.58	0.45	-0.28	0.47	0.44	0.41	0.39			
**8. OCBI**	3.52	0.83	0.42	-0.17	0.45	0.42	0.46	0.35	0.91		
**9. OCBO**	4.11	0.55	0.33	-0.35	0.31	0.30	0.17	0.30	0.76	0.42	
**10. Popularity**	3.73	0.72	0.44	-0.32	0.45	0.41	0.51	0.33	0.51	0.50	0.34

Note: All values below -0.15 and above 0.15 are statistically significant (*p* < .05)

## Discussion

The purpose of this study was to gain a deeper understanding of what it means to have well-being at work. Although scholars have long discussed what it means to have a “good life” in general [9], the workplace represents a unique context and well-being in one context does not always translate to another. To address this issue, we developed a domain-specific conceptualization and measure of eudaimonic well-being at work (EWWS). Our study included seven separate samples, 1346 participants, and multi-wave data to lend empirical support for both the EWWS and a new overall model of workplace well-being. Our results generally support our hypotheses. Specifically, we demonstrate that the EWWS can be used as a standalone scale that is distinct from general eudaimonic well-being and other similar constucts such as job engagement, life satisfaction, or leader-member exchange (Hyptheses 2, 3). Furthermore, the EWWS predicts key organizational constructs such as creativity and turnover intentions (Hypothesis 4). Importantly, our data suggest that the most complete picture of workplace well-being involves a combination of eudaimonic and hedonic perspectives (Hypothesis 5). Said simply, our work supports that well-being at work is best achieved when employees feel a two dimensional sense of meaning and purpose (intrapersonal well-being) and experience positive social interactions (interpersonal well-being; Hypotheis 1) combined with a sense of positive affect (job sastifaction) toward their roles. Finally, this overall construct of workplace well-being is significantly related to key organizational constructs such as organizational citizenship behaviors and employee popularity (Hypothesis 6).

### Theoretical implications

Our work highlights the importance of adding the interpersonal side of eudaimonic well-being at work. This is somewhat unique compared to previous conceptualizations of workplace well-being which has failed to capture the importance of workplace relationships in influencing employees’ sense of well-being at work. Although researchers have long emphasized the importance of affiliation and social needs in the workplace [86], well-being researchers have not addressed this idea directly. We suggest that the interpersonal aspects of work are particularly central to well-being in today’s modern workplaces characterized by open workspaces, virtual work, teamwork, and connective technologies that drive employee communication at work. The intrapersonal dimension of eudaimonic workplace well-being is equally important. This dimension reinforces theory that focuses on the meaningfulness and purpose employees experience when their role aligns with their deeply held values and interests. Previous work has found a positive association between an individual’s interests and his/her physical health and general well-being [87], suggesting that there may also be a link to workplace well-being.

Our findings also have implications for the study of job satisfaction. In this study, we reinforce the notion that well-being is more than just affect while still recognizing the important role played by job satisfaction. We found that when controlling for positive and negative affect, overall workplace well-being significantly predicted OCBO as well as employee popularity, whereas job satisfaction did not. This supports prior claims that workplace well-being represents more than simply affect and that it can aid scholars in explaining employee fluctuations in key organizational constructs [13].

### Practical implications

From a practical perspective, developing a sound conceptualization and measure of eudaimonic workplace well-being is important for organizations wishing to increase retention and motivation of talented employees. From our scan of the industry literature, organizations interested in workplace well-being are forced to rely on measures provided by a myriad of consulting firms whose measures often confuse concepts such as life satisfaction, happiness, and health, and employee engagement, or fail to comprehensively capture the broad conceptualization of overall workplace well-being. The classic annual “engagement survey” that is promoted by many organizations is well-known for presening a myriad of insights with little sense of how to act upon thos insights since it’s unclear what the survey is tapping. The result is that organizational decision makers do not have a clear understanding regarding what constitutes workplace well-being in the first place, how it differs from other constructs, how to ensure its reliable and valid measurement, and what do do with data that represents it.

The prominence of the interpersonal dimension of workplace well-being is also practically relevant to organizations wanting to increase well-being at work. Given the current shift from traditional workplaces, employees are engaging in less face-to-face social interaction. Approximately 30 million Americans work from home at least once a week and an estimated 3 million only work from home and these numbers are expected to grow by 63% in the next five years [88]. Despite the up-side to these non-traditional work settings in terms of decreased turnover and absenteeism [89], our findings suggests that employees’ feelings of connectedness and acceptance play an integral part in their workplace well-being. Non-traditional work environments now and in the future may not meet those needs. Future research should consider whether employees’ levels of interpersonal workplace well-being can be sufficiently satisfied virtually as online interactions continue to increase.

### Limitations and future directions

As with any study, we recognize the existence of certain limitations. First, our data was restricted to self-report data. Scholars have suggested that self-report data could be biased by any number of methodological artifacts [71]. However, since the majority of the constructs in our study centered on an individual’s subjective feelings, it is unlikely the perception of those feelings will be as accurate by others as they are by the individual him/herself. We attempted to minimize this bias by controlling for social desirability and positive and negative affect when assessing construct validity of the new eudaimonic workplace well-being measure. Future research should more closely examine whether an individual’s well-being at work can be accurately perceived and reported by others within the organization. Given the cross-sectional nature of our data collection, we cannot make causal inferences based on our study. Past research has found that the relationship between the hedonic perspective of workplace well-being (job satisfaction) and the hedonic perspective of overall well-being (subjective well-being, or happiness) is bi-directional, but the design of our study does not allow us to make such inferences [19]. Future research could make use of longitudinal data to determine if a similar bi-directional relationship exists between eudaimonic *workplace* well-being (EWWS) and eudaimonic *general* well-being (psychological well-being).

Finally, future research should also examine the relationship between an employee’s workplace well-being and the human resource practices designed to enhance well-being. For example, companies are currently spending an average of $521 per employee on corporate wellness programs designed to enhance employee well-being [90]. Future research should examine whether these programs significantly impact employee’s eudaimonic workplace well-being as well as consider which moderating factors also play an important role with regard to a wellness program’s impact. For instance, the alignment of the wellness program with the company’s overall values could help determine the ultimate impact on an employee’s eudaimonic workplace well-being. If wellness programs align with organizational values, then such alignment should spread to the employees’ interests and values and allow these programs to positively influence the intrapersonal dimension of eudaimonic workplace well-being.

### Conclusion

Current generalized measures of a person’s well-being lack comprehensiveness and the specificity required to adequately assess employee well-being at work. Hedonic workplace well-being alone fails to address areas related to meaning and purpose as well as relationships at work, which are both important in enabling employees to experience eudaimonic workplace well-being. We validate a measure of eudaimonic workplace well-being (i.e., EWWS), which reflects both intrapersonal and interpersonal dimensions of workplace well-being. This two-dimensional measure can either stand on its own as a measure of eudaimonic workplace well-being or can be combined with job satisfaction to create a three factor higher-order construct of overall workplace well-being.

## Appendix

DIRECTIONS: This portion of the survey consists of a number of statements that may describe how you feel **within your workplace**.

Please indicate your agreement with the following statements.

(1 = strongly disagree, 2 = disagree, 3 = neither agree nor disagree, 4 = agree, 5 = strongly agree)

### Interpersonal dimension

1. Among the people I work with, I feel there is a sense of brotherhood/sisterhood
2. I feel close to the people in my work environment
3. I feel connected to others within the work environment
4. I consider the people I work with to be my friends

### Intrapersonal dimension

5 I am emotionally energized at work
6 I feel that I have a purpose at my work
7 My work is very important to me
8 I feel I am able to continually develop as a person in my job

## References

[1] Saad L. The "40-Hour" Workweek is Actually Longer—by Seven Hours 2014.

[2] United States Bureau of Labor Statistics. In: Statistics USBoL, editor. 2015.

[3] Kelton. The State of Workplace Productivity Report. [Internet]. September, 2014. Available from: https://www.cornerstoneondemand.com/sites/default/files/research/csod_rs_state_of_the_workplace_2014.pdf.

[4] Briner RB, Walshe ND. An evidence‐based approach to improving the quality of resource‐oriented well‐being interventions at work. Journal of Occupational and Organizational Psychology. 2015;88(3):563–86.

[5] Sonnentag S. Dynamics of well-being. In: Morgeson FP, editor. Annual Review of Organizational Psychology and Organizational Behavior. 2015 p. 261–93.

[6] Sparks K, Faragher B, Cooper CL. Well‐being and occupational health in the 21st century workplace. Journal of occupational and organizational psychology. 2001;74(4):489–509.

[7] Wright TA, Cropanzano R, Bonett DG. The moderating role of employee positive well-being on the relation between job satisfaction and job performance. Journal of occupational health psychology. 2007;12(2):93 10.1037/1076-8998.12.2.93 17469992

[8] Culbertson SS, Fullagar CJ, Mills MJ. Feeling good and doing great: the relationship between psychological capital and well-being. Journal of occupational health psychology. 2010;15(4):421 10.1037/a0020720 21058856

[9] Ryan RM, Deci EL. On happiness and human potentials: A review of research on hedonic and eudaimonic well-being. Annual Review of Psychology. 2001;52(1):141–66. 10.1146/annurev.psych.52.1.141 11148302

[10] Diener E. Subjective well-being: The science of happiness and a proposal for a national index: American Psychological Association; 2000. 11392863

[11] Ryff CD, Keyes CLM. The structure of psychological well-being revisited. Journal of Personality and Social Psychology. 1995;69(4):719 7473027 10.1037//0022-3514.69.4.719

[12] Waterman AS. Two conceptions of happiness: Contrasts of personal expressiveness (eudaimonia) and hedonic enjoyment. Journal of Personality and Social Psychology. 1993;64(4):678.

[13] Page KM, Vella-Brodrick DA. The “what,”“why” and “how” of employee well-being: A new model. Social Indicators Research. 2009;90(3):441–58.

[14] Keyes CLM. Social well-being. Social Psychology Quarterly. 1998:121–40.

[15] Ryff CD. Happiness is everything, or is it? Explorations on the meaning of psychological well-being. Journal of Personality and Social Psychology. 1989;57(6):1069.

[16] Feldman F. Pleasure and the good life: Concerning the nature, varieties and plausibility of hedonism. Oxford: Clarendon Press; 2004.

[17] Erdogan B, Bauer TN, Truxillo DM, Mansfield LR. Whistle while you work a review of the life satisfaction literature. Journal of Management. 2012;38(4):1038–83.

[18] Jahoda M. Current concepts of positive mental health. New York, NY: Basic Books; 1958.

[19] Judge TA, Watanabe S. Another look at the job satisfaction-life satisfaction relationship. Journal of Applied Psychology. 1993;78(6):939.

[20] Keyes CLM, Shmotkin D, Ryff CD. Optimizing well-being: The empirical encounter of two traditions. Journal of Personality and Social Psychology. 2002;82(6):1007 12051575

[21] Ryff CD, Singer BH. Know thyself and become what you are: A eudaimonic approach to psychological well-being. Journal of happiness studies. 2008;9(1):13–39.

[22] Bradbury H, Lichtenstein BMB. Relationality in organizational research: Exploring the space between. Organization Science. 2000;11(5):551–64.

[23] Diener E, Wirtz D, Tov W, Kim-Prieto C, Choi D-w, Oishi S, et al New well-being measures: Short scales to assess flourishing and positive and negative feelings. Social Indicators Research. 2010;97(2):143–56.

[24] Lewis GJ, Kanai R, Rees G, Bates TC. Neural correlates of the ‘good life’: Eudaimonic well-being is associated with insular cortex volume. *Social Cognitive and Affective Neuroscience*. 2014;9(5):615–8. 10.1093/scan/nst032 23512932 PMC4014105

[25] White GB. Millennials in search of a different kind of career. The Atlantic [Internet]. 2015 Available from: http://www.theatlantic.com/business/archive/2015/06/millennials-job-search-career-boomers/395663/.

[26] Bowling NA, Eschleman KJ, Wang Q. A meta‐analytic examination of the relationship between job satisfaction and subjective well‐being. Journal of Occupational and Organizational Psychology. 2010;83(4):915–34.

[27] Hogan JR, Roberts BW. Issues and non-issues in the fidelity-bandwidth trade-off. Journal of Organizational Behavior. 1996;17(6):627–37.

[28] Pinquart MSS. 2000. Influences of socioeconomic status, social network, and competence on subjective well-being in later life: A meta-analysis. Psychology and Aging. 2000;15(2):187–224. 10879576 10.1037//0882-7974.15.2.187

[29] Judge TA, Thoresen CJ, Bono JE, Patton GK. The job satisfaction–job performance relationship: A qualitative and quantitative review. Psychological Bulletin. 2001;127(3):376 11393302 10.1037/0033-2909.127.3.376

[30] DeRue DS, Nahrgang JD, Wellman N, Humphrey SE. Trait and behavioral theories of leadership: An integration and meta‐analytic test of their relative validity. Personnel psychology. 2011;64(1):7–52.

[31] Schaufeli WB, Bakker AB. Job demands, job resources, and their relationship with burnout and engagement: A multi-sample study. Journal of Organizational Behavior. 2004;25(3):293–315.

[32] Kahn. Psychological conditions of personal engagement and disengagement at work. Academy of Management Journal. 1990;33(4):692–724.

[33] Bradburn NM. The structure of psychological well-being. Oxford, England: Aldine; 1969.

[34] Diener E, Lucas RE. Personality and subjective well-being. Well-being: Foundations of Hedonic Psychology 1999:213.

[35] Andrews FM, Withey SB. Measuring global well-being Social Indicators of Well-Being: Springer; 1976 p. 63–106.

[36] Duffy MK, Ganster DC, Pagon M. Social undermining in the workplace. Academy of Management Journal. 2002;45(2):331–51.

[37] Kasser VG, Ryan RM. Mortality in a Nursing Home. Journal of Applied Social Psychology. 1999;29(5):935–54.

[38] Amabile TM. Children's artistic creativity detrimental effects of competition in a field setting. Personality and Social Psychology Bulletin. 1982;8(3):573–8.

[39] Ryan RM, Deci EL. Self-determination theory and the facilitation of intrinsic motivation, social development, and well-being. American Psychologist. 2000;55(1):68 11392867 10.1037//0003-066x.55.1.68

[40] Hobfoll SE. Conservation of resources theory: Its implication for stress, health, and resilience In: Folkman S, editor. The Oxford Handbook of Stress, Health, and Coping. New York, NY: Oxford University Press; 2011 p. 127–47.

[41] George JM, Jones GR. The experience of work and turnover intentions: interactive effects of value attainment, job satisfaction, and positive mood. Journal of Applied Psychology. 1996;81(3):318 8690691 10.1037/0021-9010.81.3.318

[42] Alarcon GM, Edwards JM. The relationship of engagement, job satisfaction and turnover intentions. Stress and Health. 2011;27(3):e294–e8.

[43] Nix GA, Ryan RM, Manly JB, Deci EL. Revitalization through self-regulation: The effects of autonomous and controlled motivation on happiness and vitality. Journal of Experimental Social Psychology. 1999;35(3):266–84.

[44] Urry HL, Nitschke JB, Dolski I, Jackson DC, Dalton KM, Mueller CJ, et al Making a life worth living: Neural correlates of well-being. Psychological Science. 2004;15(6):367–72. 10.1111/j.0956-7976.2004.00686.x 15147488

[45] Hinkin TR. A brief tutorial on the development of measures for use in survey questionnaires. Organizational Research Methods. 1998;1(1):104–21.

[46] Ilies R, Judge TA. On the heritability of job satisfaction: The mediating role of personality. Journal of Applied Psychology.88(4):750–9. 12940413 10.1037/0021-9010.88.4.750

[47] Organ DW. Organizational citizenship behavior: The good soldier syndrome. Washington D.C: Lexington Books; 1988.

[48] Organ DW, Podsakoff PM, Podsakoff NP. Expanding the criterion domain to include organizational citizenship behavior: Implications for employee selection In: Zedeck S, editor. APA Handbooks in Psychology. 8 Washington D.C: American Psychological Association; 2011.

[49] Scott BA, Judge TA. The popularity contest at work: Who wins, why, and what do they receive? Journal of Applied Psychology. 2009;94(1):20 10.1037/a0012951 19186893

[50] Van Zelst RH. Empathy test scores of union leaders. Journal of Applied Psychology. 1952;36(5):293.

[51] Koopman J, Matta FK, Scott BA, Conlon DE. Ingratiation and popularity as antecedents of justice: A social exchange and social capital perspective. Organizational Behavior and Human Decision Processes. 2015;131:132–48.

[52] Anderson JC, Gerbing DW. Structural equation modeling in practice: A review and recommended two-step approach. Psychological Bulletin. 1988;103(3):411.

[53] Hinkin TR, Tracey JB. An analysis of variance approach to content validation. Organizational Research Methods. 1999;2(2):175–86.

[54] Ferris DL, Brown DJ, Berry JW, Lian H. The development and validation of the workplace ostracism scale. Journal of Applied Psychology. 2008;93(6):1348 10.1037/a0012743 19025252

[55] Mirowsky J, Ross CE. Meaningful comparison versus statistical manipulation: A Reply to Johnson and Benin. American Journal of Sociology. 1984;89(5):1194–200.

[56] Ryff CD. Psychological well-being in adult life. Current Directions in Psychological Science. 1995:99–104.

[57] Rich BL, LePine JA, Crawford ER. Job engagement: Antecedents and effects on job performance. Academy of Management Journal. 2010;53(3):617–35.

[58] Diener E, Emmons RA, Larsen RJ, Griffin S. The satisfaction with life scale. Journal of Personality Assessment. 1985;49(1):71–5. 10.1207/s15327752jpa4901_13 16367493

[59] Liden RC, Maslyn JM. Multidimensionality of leader-member exchange: An empirical assessment through scale development. Journal of Management. 1998;24(1):43–72.

[60] Oldham GR, Cummings A. Employee creativity: Personal and contextual factors at work. Academy of Management Journal. 1996;39(3):607–34.

[61] Hom PW, Griffeth RW. Structural equations modeling test of a turnover theory: Cross-sectional and longitudinal analyses. Journal of Applied Psychology. 1991;76(3):350.

[62] Scott KD, Taylor GS. An examination of conflicting findings on the relationship between job satisfaction and absenteeism: A meta-analysis. Academy of Management Journal. 1985;28(3):599–612.

[63] Diener E, Seligman ME. Beyond money toward an economy of well-being. Psychological Science in the Public Interest. 2004;5(1):1–31. 10.1111/j.0963-7214.2004.00501001.x 26158992

[64] Kahneman D, Diener E, Schwarz N. Well-being: Foundations of hedonic psychology: Russell Sage Foundation; 1999.

[65] Nelson A, Cooper CL, Jackson PR. Uncertainty amidst change: The impact of privatization on employee job satisfaction and well‐being. Journal of Occupational and Organizational psychology. 1995;68(1):57–71.

[66] Weiss HM. Deconstructing job satisfaction: Separating evaluations, beliefs and affective experiences. Human Resource Management Review. 2002;12(2):173–94.

[67] Hackman J, Oldman G. The job diagnostic survey: An instrument for the diagnostis of job redesign projects. New Haven, CT: Yale University; 1974.

[68] Williams LJ, Anderson SE. Job satisfaction and organizational commitment as predictors of organizational citizenship and in-role behaviors. Journal of Management. 1991;17(3):601–17.

[69] Gallagher MW, Lopez SJ, Preacher KJ. The Hierarchical Structure of Well‐Being. Journal of Personality. 2009;77(4):1025–50. 10.1111/j.1467-6494.2009.00573.x 19558444 PMC3865980

[70] Watson D, Clark LA, Tellegen L. Development and validation of brief measures of positive and negative affect: the PANAS scales. Journal of Personality and Social Psychology. 1988;54(6):1063 3397865 10.1037//0022-3514.54.6.1063

[71] Podsakoff PM, MacKenzie SB, Lee J-Y, Podsakoff NP. Common method biases in behavioral research: A critical review of the literature and recommended remedies. Journal of Applied Psychology. 2003;88(5):879–903. 10.1037/0021-9010.88.5.879 14516251

[72] Paulhus DL. Measurement and control of response bias In: Robinson JP, Shaver PR, Wrightsman LS, editors. Measures of social psychological attitudes. 1. San Diego, CA: Academic Press; 1991 p. 17–59.

[73] Porath C, Spreitzer G, Gibson C, Garnett FG. Thriving at work: Toward its measurement, construct validation, and theoretical refinement. Journal of Organizational Behavior. 2012;33(2):250–75.

[74] Hu LT, Bentler PM. Cutoff criteria for fit indexes in covariance structure analysis: Conventional criteria versus new alternatives. Structural Equation Modeling: A Multidisciplinary Journal. 1999;6(1):1–55.

[75] Cortina JM. What is coefficient alpha? An examination of theory and applications. Journal of Applied Psychology. 1993;78(1):98.

[76] Cronbach LJ, Meehl PE. Construct validity in psychological tests. Psychological Bulletin. 1955;52(4):281 13245896 10.1037/h0040957

[77] Kerlinger FN, Lee HB. Foundations of behavioral research: Educational and psychological inquiry. New York: Holt, Rinehart and Winston; 1964.

[78] Hoyle RH. Handbook of Structural Equation Modeling: Guilford Publications; 2014.

[79] Anderson JC, Gerbing DW. Predicting the performance of measures in a confirmatory factor analysis with a pretest assessment of their substantive validities. Journal of Applied Psychology. 1991;76(5):732.

[80] Fornell C, Larcker DF. Evaluating structural equation models with unobservable variables and measurement error. Journal of Marketing Research. 1981:39–50.

[81] Marsh HW, Hocevar D. Application of confirmatory factor analysis to the study of self-concept: First-and higher order factor models and their invariance across groups. Psychological Bulletin. 1985;97(3):562.

[82] Rees MJ, Earles JA. Intelligence is the best predictor of job performance. Current Directions in Psychological Science. 1992:86–9.

[83] Colquitt JA. On the dimensionality of organizational justice: A construct validation of a measure. Journal of Applied Psychology. 2001;86(3):386 11419799 10.1037/0021-9010.86.3.386

[84] Múthen, Múthen. MPlus. 7 ed2014.

[85] Hoffman BJ, Blair CA, Meriac JP, Woehr DJ. Expanding the criterion domain? A quantitative review of the OCB literature. Journal of Applied Psychology. 2007;92(2):555 10.1037/0021-9010.92.2.555 17371100

[86] Maslow AH. A theory of human motivation. Psychological Review. 1943;50(4):370–96.

[87] Spokane AR, Meir EI, Catalano M. Person–environment congruence and Holland's theory: A review and reconsideration. Journal of Vocational Behavior. 2000;57(2):137–87.

[88] Rapoza K. One in five Americans work from home, numbers seen rising over 60%. Forbescom [Internet]. 2013.

[89] Golden TD, Veiga JF, Simsek Z. Telecommuting's differential impact on work-family conflict: Is there no place like home? Journal of Applied Psychology. 2006;91(6):1340 10.1037/0021-9010.91.6.1340 17100488

[90] Emerman E. New Health Care Survey Finds Spending on Wellness Initiatives Has Doubled in the Last Four Years. 2013.

